# Pharmacological inhibition of endoplasmic reticulum stress mitigates osteoporosis in a mouse model of hindlimb suspension

**DOI:** 10.1038/s41598-024-54944-7

**Published:** 2024-02-27

**Authors:** Hiba Al-Daghestani, Rizwan Qaisar, Sausan Al Kawas, Nurhafizah Ghani, K. G. Aghila Rani, Muhammad Azeem, Hijaz Kamal Hasnan, Nur Karyatee Kassim, A. R. Samsudin

**Affiliations:** 1 Department of Oral and Craniofacial Health Sciences, College of Dental Medicine, University of Sharjah, Sharjah, 27272 UAE; 2 Department of Basic Medical Sciences, College of Medicine, University of Sharjah, Sharjah, 27272 UAE; 3 Space Medicine Research Group, Research Institute for Medical and Health Sciences, University of Sharjah, Sharjah, 27272 UAE; 4 School of Dental Sciences, Health Campus, Universiti Sains Malaysia, Kubang Kerian 16150, Kelantan, Malaysia; 5 Research Institute for Medical and Health Sciences, University of Sharjah, Sharjah, 27272 UAE; 6 Department of Mathematical and Physical Sciences, University of Nizwa, Nizwa 33, Sultanate of Oman; 7 Department of Geology, Faculty of Science, University of Malaya, Kuala Lumpur, Malaysia

**Keywords:** 4-PBA, ER stress, HLS mice, Osteoporosis, Bone resorption, Biological techniques, Molecular biology, Pathogenesis, Chemistry

## Abstract

Hindlimb suspension (HLS) mice exhibit osteoporosis of the hindlimb bones and may be an excellent model to test pharmacological interventions. We investigated the effects of inhibiting endoplasmic reticulum (ER) stress with 4-phenyl butyrate (4-PBA) on the morphology, physicochemical properties, and bone turnover markers of hindlimbs in HLS mice. We randomly divided 21 male C57BL/6J mice into three groups, ground-based controls, untreated HLS group and 4-PBA treated group (HLS+4PBA) (100mg/kg/day, intraperitoneal) for 21 days. We investigated histopathology, micro-CT imaging, Raman spectroscopic analysis, and gene expression. Untreated HLS mice exhibited reduced osteocyte density, multinucleated osteoclast-like cells, adipocyte infiltration, and reduced trabecular striations on micro-CT than the control group. Raman spectroscopy revealed higher levels of ER stress, hydroxyproline, non-collagenous proteins, phenylalanine, tyrosine, and CH_2_Wag as well as a reduction in proteoglycans and adenine. Furthermore, bone alkaline phosphatase and osteocalcin were downregulated, while Cathepsin K, TRAP, and sclerostin were upregulated. Treatment with 4-PBA partially restored normal bone histology, increased collagen crosslinking, and mineralization, promoted anti-inflammatory markers, and downregulated bone resorption markers. Our findings suggest that mitigating ER stress with 4-PBA could be a therapeutic intervention to offset osteoporosis in conditions mimicking hindlimb suspension.

## Introduction

Osteoporosis is one of the most common skeletal metabolic diseases and is characterized by a reduction of bone mass and alteration of bone micro-architecture leading to increased bone fragility and fracture risk. The economic burden on the therapeutics of osteoporotic fractures is enormous since osteoporosis affects over 200 million women worldwide, and causes 8.9 million fractures annually^1^. Mortality rates for osteoporotic fractures range from 15 to 30%, which is comparable to stroke and breast cancer^2^. The key mechanisms of osteoporosis development are decreased osteoblast function and increased osteoclast activity^3^. This cellular pathology arises from multifactorial conditions including hormonal factors, dysregulation in endocrine metabolism, reduced absorption of calcium, and an unhealthy lifestyle. The endoplasmic reticulum (ER) is considered as the protein-folding factory of cells. It is responsible for biosynthesis, folding, assembly, and modification of proteins^4^. A stressful environment, such as microgravity conditions, can disrupt ER function in many cells by accumulating unfolded and misfolded proteins in the ER lumen, resulting in ER stress^5,6^. To restore homeostasis and reduce stress, the ER activates a signaling network known as the unfolded protein response (UPR)^7^. Recent studies have found that many diseases, such as cancer, inflammation, neurodegeneration, and osteoporosis, are related to excessive ER stress^8^. Additionally, ER stress, oxidative stress, autophagy, and apoptosis are closely linked^9^. Ultimately, chronic unresolved ER stress leads to apoptosis through UPR^8,10^.

There are three well-established animal models for studying osteoporosis: the immobilization-induced bone loss model, the ovariectomized model, and the glucocorticoid–induced osteoporosis model. These models shed light on the experimental comparison of the osteoporosis stages in humans^11^. Increased apoptotic death of osteoblasts is observed in both glucocorticoid-induced and postmenopausal osteoporosis in mice studies, providing insight into the link between osteoporosis pathogenesis and ER stress in osteoblasts^12^. Some ER stress markers especially Glucose-Regulated Protein 78 (GRP78), C/EBP homologous protein (CHOP), and transcription factor X-box-binding protein-1 (XBP-1) are upregulated in apoptotic osteoblasts, indicating an ER stress implication in osteoporosis^13^.

Immobilization-induced bone loss model is one of the commonly studied animal models of osteoporosis to study the effect of disuse on the musculoskeletal system^14^. This model can be created in mice either by tail suspension or by hindlimb immobilization via sciatic neurectomy, botulinum toxin paralysis, or using a plaster cast that induces osteoporosis in cortical and trabecular bones due to skeletal disuse^15^. Hindlimb suspension (HLS) is a well-established model to replicate the effects of prolonged bed rest and space microgravity on the musculoskeletal system in rodents^16^.

The molecular pathogenesis of disuse osteoporosis is not fully understood, and ER stress may contribute to disuse-induced bone loss in the HLS mice. While ER stress has been shown to stimulate osteoblast apoptosis, increase bone loss, and promote osteoporosis development^17^, recent *in-vivo* studies have demonstrated the role of 4-phenyl butyrate (4-PBA) in improving bone phenotypes in certain bone diseases that involve disturbance of bone matrix such as Osteogenesis Imperfecta in the mouse model^18^.

4-PBA inhibits ER stress by regulating several vital proteins, including GRP78, PKR-like ER kinase (PERK), c-Jun N-terminal kinase (c-JNK), XBP-1, and CHOP^19^. It can reduce intracellular misfolded proteins via a homeostatic signaling network by regulating several vital proteins, thereby reducing ER stress^20^. Other authors have previously demonstrated that suppression of ER stress using 4-PBA in the HLS model has effectively mitigated skeletal muscle loss^21^, but the role of 4-PBA in mitigating bone loss is not yet established. Thus, suppressing ER stress with the usage of 4-PBA may be an appealing molecular intervention to prevent osteoporosis in HLS mice. This study aimed to compare the histopathological changes, physicochemical properties, and bone turnover markers of HLS-induced osteoporotic femur and tibia of HLS mice treated with and without 4-PBA.

## Materials and methods

### Animal preparation

Male C57BL/6J mice, four months old, and weighing 25–30 g, were used in this study. This study was approved by the University of Sharjah Animal Care and Use Committee, (ACUC- 19–05-05–01) along with the Universiti Sains Malaysia Institutional Animal Care and Use Committee (USM IACUC; USM/IACUC/2022/(135) (1192). All procedures conducted agreed with accepted international standards, and all methods were carried out in accordance with the relevant guidelines and regulations of both institutional ethics committees. Additionally, all methods and results were reported in accordance with ARRIVE guidelines^22^. Mice were placed in specialized cages (one cage per mouse) in the animal facility at the University of Sharjah, Sharjah, United Arab Emirates, under standard laboratory conditions at a steady temperature of 24 °C ± 1 and humidity at 55% ± 5. The light/dark cycle was 12 h, with lights on from 8:00 a.m. to 8:00 p.m. Mice had free access to water and maintenance diet (Altromin Spezielfutter GmbH & Co) ad libitum.

### Experimental design

A total of 21 male C57BL/6J mice (Charles River, Italy), were randomly divided into three groups of seven mice each and placed in similar housing conditions. Group I consisted of freely moving ground-based control mice, group II underwent HLS (untreated HLS) and group III underwent HLS with daily intraperitoneal injections of 4-PBA (HLS + 4-PBA) (Santa Cruz Biotechnology, CA, USA), at 100mg/kg/d^23^. The duration of HLS was 21 days^24^. Hindlimb unloading was performed by tail suspension based on previously reported methods^14^. Briefly, mice were placed individually in a 46 × 46 × 46-cm cage equipped with a pulley at the top. The tails were cleaned prior to string application from the tail to a metal rod at the top of the specialized cage. The tape was adjusted to suspend the hindlimbs away from the ground at approximately 30-degree head tilt. The forelimbs were weight-bearing and remained in constant contact with the ground, enabling movement of the mice in the cage, and allowing freedom to access food and water throughout the suspension period. The animals were reviewed daily to ensure that their hindlimbs were not touching the ground and were free from pain. General health, activity, responsiveness, and appearance were also assessed daily.

At the end of the 21-day test period, mice from all groups were euthanized by cervical dislocation. Harvesting of bilateral hindlimbs (femur and tibia), and forelimbs (humerus) was performed. The bones were carefully cleaned off from soft tissues and stored at -80 °C for further use or placed in respective buffers/culture medium for reactive oxygen species (ROS) assay, histopathological assessment, micro-CT imaging, Raman spectroscopic analysis, and gene expression studies. The flowchart of the study design is shown in supplementary Fig. 1.

### Reactive oxygen species assay

ROS generation was measured for hindlimbs (femur and tibia) and compared with forelimbs (humerus) in ground control (n = 3/group) and untreated HLS (n = 3/group) groups. Briefly, following euthanasia, the bone marrow cells were harvested by flushing hindlimbs, and forelimbs obtained from ground control and untreated HLS groups, using phosphate-buffered saline (PBS). The cells were incubated at a density of 5–10 × 10^5^ cells with RBS lysis buffer at room temperature for 10 min with frequent mild vertexing to remove contaminating red blood cell population. The cells were then washed in PBS and resuspended with 250 µL of ROS assay buffer containing 2µl of fluorogenic ROS stain (Invitrogen, USA). The cell suspension was then transferred into flow cytometry tubes and incubated at 37 °C in a humidified chamber for 60 min. Following incubation, ROS levels were estimated by flow cytometry in the FITC channel at 520 nm wavelength using the BD FACS Aria III cell sorter (Beckton D Biosciences, United Kingdom). Data of average intensity were analyzed using the FlowJo software program and expressed as a percent increase in ROS generation adjusted for ground controls.

### Histopathological & histomorphometric studies

Hindlimbs of all groups were subjected to histomorphometric studies. The left tibia (n = 3/group) was cleaned from soft tissues and fixed in 10% formalin for two days. The bone samples underwent decalcification using chelating agent 10% ethylenediaminetetraacetic acid (EDTA) (EMD Millipore Corporation, Billerica, MA, USA), at pH 7.4, for seven weeks in a 4 °C cold room with continuous shaking and renewal of EDTA every week, until the bones turned soft. After decalcification, samples were sectioned by a semi-automated rotary microtome (Leica Biosystems RM2245, Germany) at 5 µm thickness. The sections were further subjected to hematoxylin and eosin stain (H&E) (Sigma-Aldrich) using standard protocols. An upright microscope, Olympus BX43 (Olympus Optical, Tokyo, Japan), equipped with a digital camera (Olympus, DP 74) was used to evaluate the slides. The images were then exported as .tiff files for quantification using Image J (National Institute of Health, Bethesda, MD, USA). Region of interest (ROI) in tibia was identified at the diaphysis region and proximal tibial metaphysis at the primary spongiosa, located distal to the growth plate. The nomenclature of histological parameters used was based on the American Society for Bone and Mineral Research's specified standards for structural, dynamic, and cellular parameters^25^. The number of osteocytes (N.Ot) in lacunae per square millimeter was counted manually at the diaphysis region in the cortical bone among the three study groups, while the number of adipocytes (N.Ad) cells per square millimeter was counted in the bone marrow area.

### Micro- computed tomography (micro-CT) analysis

Microarchitecture for trabecular and cortical bone was examined by micro-CT. The collected tibias from all study groups (n = 3/group) were washed with phosphate-buffered saline (PBS) and fixed with 4% paraformaldehyde. The bones were scanned with a high-resolution ZEISS Xradia Versa XRM -520 CT (Carl Zeiss XRM, Pleasanton, CA, USA) 3D X-ray microscope following the guidelines recommended by Bouxsein and colleagues^26^. Samples were placed in foam mold, and images were acquired at rodent bone settings 80kV/7W power energy, using a 0.5-mm aluminum filter with an isotropic voxel size of 11 μm. All scans had reconstruction parameters applied identically. The micro-CT data was exported in DICOM format. Three-dimensional reconstruction and bone analysis parameters were done using DragonFly ORS software (version 2022.2.0.1227 for Windows 10; Object Research System (ORS), Montreal, Canada).

A normalizing filter was used to distribute subject-specific grayscale histograms uniformly. The representative gross specimen and its two-dimensional images were arranged with their respective anatomical views, namely, gross specimen, anterior, axial, and cortical cross-section. Image segmentation and further subdivision into specific ROI were done manually. ROI in the tibia was chosen based on published literature^27,28^, specifically, at the proximal and distal metaphysis for trabecular analysis and diaphysis of tibia for the cortical bone analysis. Manual segmentation of the ROI region was performed with green color used for cortical bone segmentation, and purple color was used for trabecular bone segmentation.

Trabecular bone volume fraction (BV/TV,%), trabecular thickness (Tb.Th, mm), and trabecular separation (Tb.Sp, μm) were measured. The trabecular number (Tb.N, 1/mm) was calculated according to 3D measurements for the spacing of trabeculae using the following formula^29^: $Tb.N=\frac{1}{Tb.Th+Tb.Sp}$.

Structural anisotropy, analyzed by mean intercept length (MIL) was used to describe the orientation of structural elements. Cortical thickness (Ct. Th, mm^2^), total cross-sectional cortical area (Tt. Ar, mm^2^), and cortical bone area (Ct. Ar, mm^2^) were also measured.

### Physicochemical analysis by Raman spectroscopy

Femur bones (n = 5/group) were placed on glass slides and analyzed using inVia Renishaw Raman Microscope (London, United Kingdom) equipped with a 785 nm wavelength edge laser operated with a 20 × objective lens and a laser power of 1% and spot size of 50 μm with an approximate 1 cm distance between the probe and the samples to provide an appropriate focus. The spectral range was from 100 cm^−1^ to 3000 cm^−1^, to identify the signature peaks from the biological molecules^30^. For each study group, five locations were analyzed along the diaphysis of the femur. The average of the readings was taken with an integration time of 30 s. Average Raman spectra graph plotting was performed using OriginPro, Version 2022b (OriginLab Corporation, Northampton, MA, USA). The peaks spectra were normalized to the intensity of the phosphate (v_1_PO_4_^–3^).

Distinctive Raman peaks pertaining to the vibrational modes of ER stress markers were identified at (1443 cm^−1^ and 1654 cm^−1^)^31^. Quantitative analysis of collagen matrix components (organic) was assigned to amide I (1660 cm^−1^ and 1690 cm^−1^), amide III (1242 cm^−1^), CH_2_Wag (1450 cm^−1^), proline (855 cm^−1^), and hydroxyproline (876 cm^−1^), while mineral components were assigned to phosphate peaks *v*_1_PO_4_ at (960 cm^−1^) and *v*_2_PO_4_ at (430 cm^−1^) and carbonate peak v_1_CO_3_ at (1070 cm^−1^). Amino acids related to bone composition were measured, namely, phenylalanine (1003 cm^−1^), tyrosine (1204 cm^−1^), proteoglycan (1375 cm^−1^), and adenine (1325 cm^−1^)^32–34^. To determine mineral-to-matrix composition, ratios of mineral/proline + hydroxyproline and *v*_2_ phosphate/amide III were calculated. Carbonate to phosphate substitution ratio (v_1_CO_3_^2−^/*v*_2_PO_4_^3^), and amide I/amide I, depicting collagen crosslink maturity were also analyzed^35^.

### Gene expression studies

Total RNA was extracted from the hindlimbs (femur and tibia) of animals from all groups (n = 3/group) using the RNeasy kit (Invitrogen, USA). The quality of the RNA samples was measured using the NanoDrop ND1000 (Thermo Fisher Scientific, USA). RNA was reverse transcribed to cDNA using SuperScript™ II reverse transcriptase kit (Invitrogen, USA). The expression of osteoblastic, osteoclastic, and osteocytic markers was quantified by real-time PCR using 5X FIREPOL SYBRGreen Mix (Solisbiodyne, Estonia). Primers used include alkaline phosphatase (ALP), osteocalcin (OC), tartrate-resistant acid phosphatase (TRAP), Cathepsin K (Cat K), and Sclerostin. 18S was used as an internal control for data normalization. The primers used for the reactions are given in Supplementary Table 1. The qPCR amplification was conducted with an initial denaturation at 95 °C for 10 min, followed by 40 cycles at 95 °C for 30 s, 60 °C for 1 min, and 72 °C for 1 min. The reaction was performed using StepOne Thermocycler (Applied Biosystems, USA), and the results were quantified using the ΔΔCt relative quantification method.

### Statistical analysis

Statistical analysis was carried out using GraphPad Prism version 8 software (GraphPad Software, La Jolla, CA). All numerical results were reported as mean ± SEM, and group comparisons were performed using one-way ANOVA and Tukey's multiple comparison tests. *p* < 0.05 was considered statistically significant.

## Results

### 4-PBA did not affect the morphology and body weights of HLS mice

A total of 21 male C57BL/6J mice, were included in the study, and all animals completed the 21 days HLS period. There was no failure of tail suspension, and none of the animals were injured during the study. All animals in untreated HLS and HLS + 4-PBA groups remained freely mobile on their forelimbs and were able to feed themselves, possessed a healthy coat, and remained active throughout the study period. Despite these clinical observations, both untreated HLS and HLS + 4-PBA group showed a significant drop in body weight compared to the control (*p* < 0.05). In contrast, HLS + 4-PBA group that received treatment with 4-PBA demonstrated a slightly higher body weight compared to untreated HLS (supplementary Fig. 2).

### HLS increased the ROS production in hindlimbs

Results showed that a high level of ROS generation in both the hindlimbs and forelimbs of untreated HLS group compared to controls. When the hindlimbs were compared with the forelimbs, a significantly higher ROS generation was observed in the hindlimbs than in the forelimbs of the untreated HLS group (*p* < 0.0001) (supplementary Fig. 3).

### 4-PBA inhibited the HLS-induced disruption of bone histology

**Figure 1:**
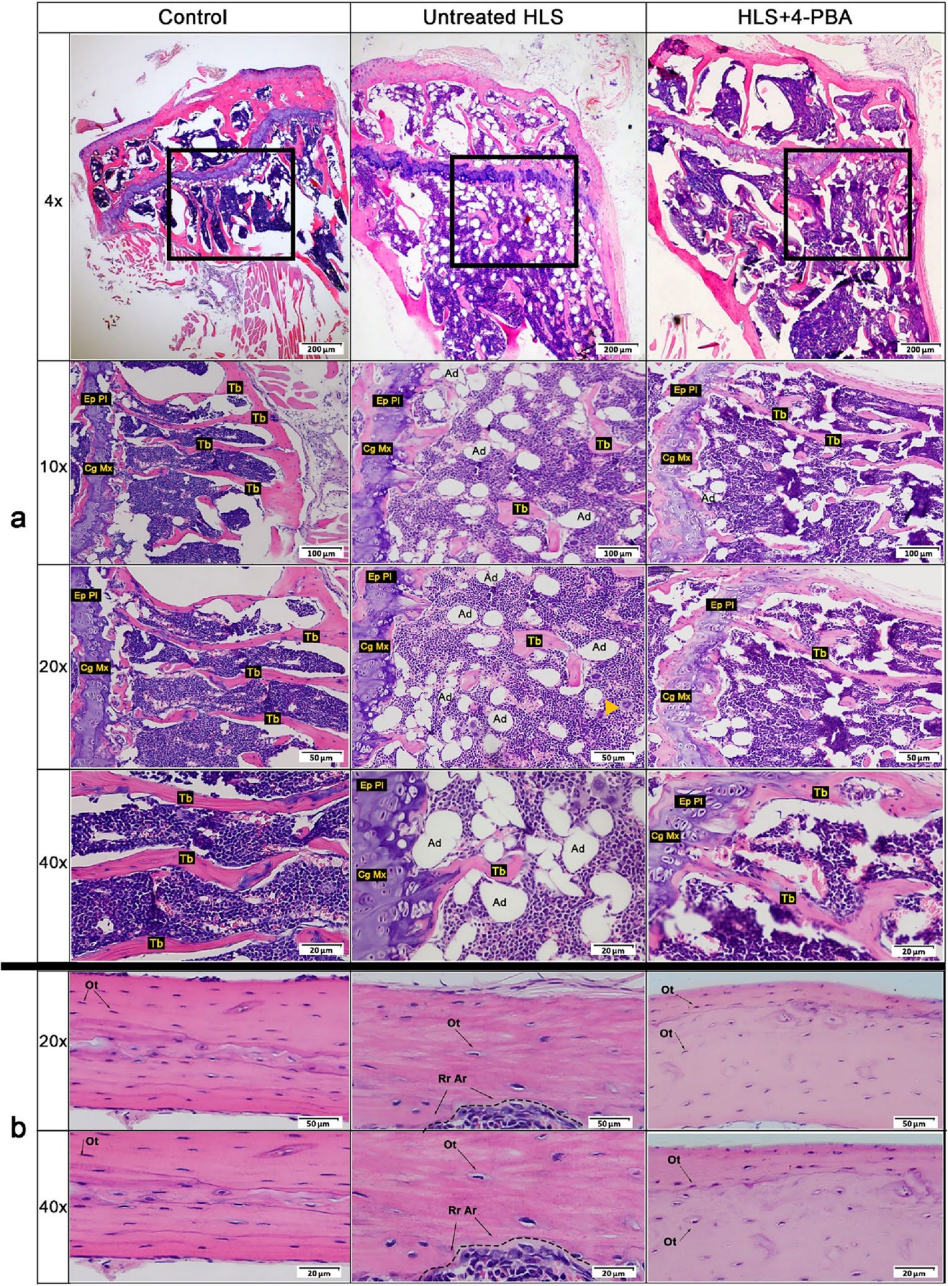
Representative histological sections of trabecular bone of proximal tibia and cortical bone at the diaphysis of the tibia. (**a**) H&E staining of decalcified longitudinal histological section of trabecular bone. Comparisons among the control, untreated HLS, and HLS + 4-PBA treated groups are shown at 4x, 10x, 20x, and 40 × magnification. (*Arrowhead*) point to multinucleated giant cells (MGC) resembling osteoclast progenitor cells in the bone marrow found throughout the untreated HLS group. Adipocytes (Ad) and cartilage matrix (Cg Mx) in the epiphyseal plate are labeled in the sections. (**b**) H&E staining of decalcified longitudinal histological section of cortical bone. Comparisons among the control, untreated HLS, and HLS + 4-PBA treated groups are shown at 20x, and 40 × magnification. (n = 3/group) (HLS; hindlimb suspension, 4-PBA; 4-phenyl butyrate, H&E; Haematoxylin and Eosin, Tb; Trabecular bone, Ep Pl; epiphyseal plate, MGC; multinucleated giant cells, Ad; adipocytes, Cg Mx; cartilage matrix, Ot; Osteocytes, Rr Ar; Resorption area).

Histological features of bone trabeculae of the proximal tibia were examined and compared among the three study groups (Fig. 1). In the untreated HLS group, the presence of cracks and moderately thin trabeculae that lacked connectivity, together with a marked increase in trabecular separation and a significant reduction in the number of trabeculae that are often missing in some areas was noted; as compared to the structure of normal proximal tibia in the control group that showed typical dense, uniform, and unbroken bone trabeculae structures. Untreated HLS group also showed the presence of multinucleated giant cells (MGC) (pointed with arrowheads) resembling osteoclast progenitor cells found throughout the bone marrow (Fig. 1). In the HLS + 4-PBA group, there was a relatively higher number of trabeculae and less trabecular separation with a more uniform continuity of the trabeculae plates similar to the control group. A smaller number of multinucleated giant cells resembling osteoclast were found in the HLS + 4-PBA group. The number of adipocyte cells in the untreated HLS group was much higher when compared with the other two groups, as shown in (Fig. 1). Chondrocytes were also observed in the epiphyseal plate, which appeared less organized in the untreated HLS group compared to HLS + 4-PBA and the control groups.

Histological features of the bone cortex at the diaphysis of the tibia in H&E staining were examined and compared among the three groups (Fig. 1). The untreated HLS group demonstrated significantly less number of osteocytes (less cellular) compared to the control group (*p* < 0.001; supplementary Fig. 4b), while HLS + 4-PBA group showed the presence of a significantly higher number of osteocytes (more cellular) compared to the untreated HLS (*p* < 0.05; supplementary Fig. 4b). Nevertheless, the cellular density observed in HLS + 4-PBA-treated group remains lower than that in the control group. Untreated HLS group also showed the presence of resorption areas within the cortex (demarcated), while this feature is not found in HLS + 4-PBA-treated group.

### 4-PBA inhibited the HLS-induced disruption of bone microarchitecture

**Figure 2:**
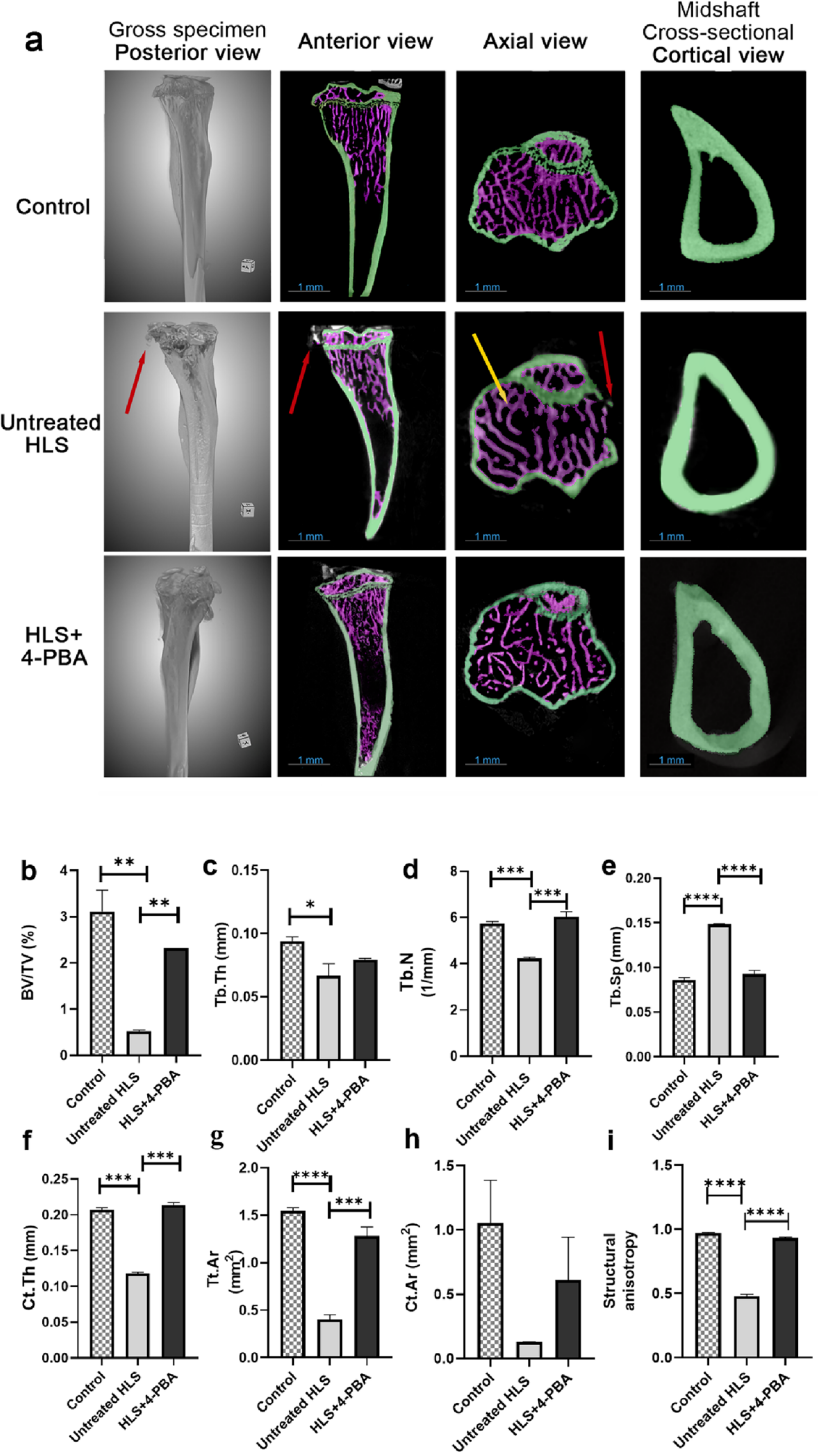
Representation of tibiae gross morphology and micro-CT analysis of trabecular and cortical structural parameters among control, untreated HLS, and HLS + 4-PBA groups. (**a**) Representative photos of tibiae from the three study groups in posterior, anterior, axial, and cross-sectional cortical views. In all image panels, the green color represents segmented cortical bone, and the purple color represents trabecular bone segmentation using DragonFly ORS software. The upper panel represents the control group; the middle panel represents the untreated HLS group, and the lower panel represents the HLS + 4-PBA group. The upper panel demonstrated the micro-image of the tibia, showing a well-demarcated cortical outline (in green) with normal trabecula striae (in purple). The middle panel demonstrated loss of outer cortical outline (pointed by red arrow) of the tibia associated with an increase in trabecular separations (increased spaces between the striae, pointed by yellow arrow in axial view) in untreated HLS. The lower panel demonstrated a normal cortical outline and adequate density of trabecular striae resembling images seen in the control group. (**b**) Bone volume fraction (BV/TV%), (**c**) Trabecular thickness (Tb.Th) (mm), (**d**) Trabecular number (Tb.N) (1/mm), (**e**) Trabecular separation (Tb.Sp) (mm), (**f**) Cortical thickness (Ct.Th) (mm), (**g**) Total cross-sectional of cortical area (Tt.Ar) (mm^2^), (**h**) Cortical bone area (Ct.Ar) (mm^2^), and (**i**) structural anisotropy. Data are expressed as mean ± SEM. (n = 3/group) (HLS; hindlimb suspension, 4-PBA; 4-phenyl butyrate). (*p < 0.05; **p < 0.01; ***p < 0.001; ****p < 0.0001).

Micro-CT quantitative analysis for trabecular and cortical bone parameters was compared among the study groups. Representative images are shown from each group in Fig.  2, with two-dimensional images arranged with their respective anatomical views, namely, gross specimen, anterior, axial, and cortical cross section (Fig. 2a). The mice in the untreated HLS group showed a significant decrease in bone volume fraction (BV/TV,%) than both the control and HLS + 4-PBA groups (*p* < 0.01; Fig. 2b). HLS + 4-PBA treated group had higher BV/TV than untreated HLS Group (*p* < 0.01; Fig. 2b). The trabecular compartment was reduced in the untreated HLS group as shown by significant reductions in trabecular thickness (*p* < 0.05; Fig. 2c) and trabecular striations number (*p* < 0.001; Fig. 2d). This resulted in a significant increase in the trabecular separation in the untreated HLS group compared to the control and HLS + 4-PBA group (*p* < 0.0001; Fig. 2e). The cortical compartment followed similar trends in the untreated HLS group with a significant reduction in cortical thickness (*p* < 0.001; Fig. 2f) and total cross-sectional area (*p* < 0.0001; Fig. 2g), and less cortical bone area (Fig. 2h). The degree of anisotropy demonstrating the structural element pattern was calculated, and the untreated HLS group had a significantly lower degree of anisotropy when compared to control and HLS + 4-PBA treated group *p* < 0.0001; Fig. 2i).

### 4-PBA partly prevented the alterations in Raman spectroscopy of HLS mice

**Figure 3:**
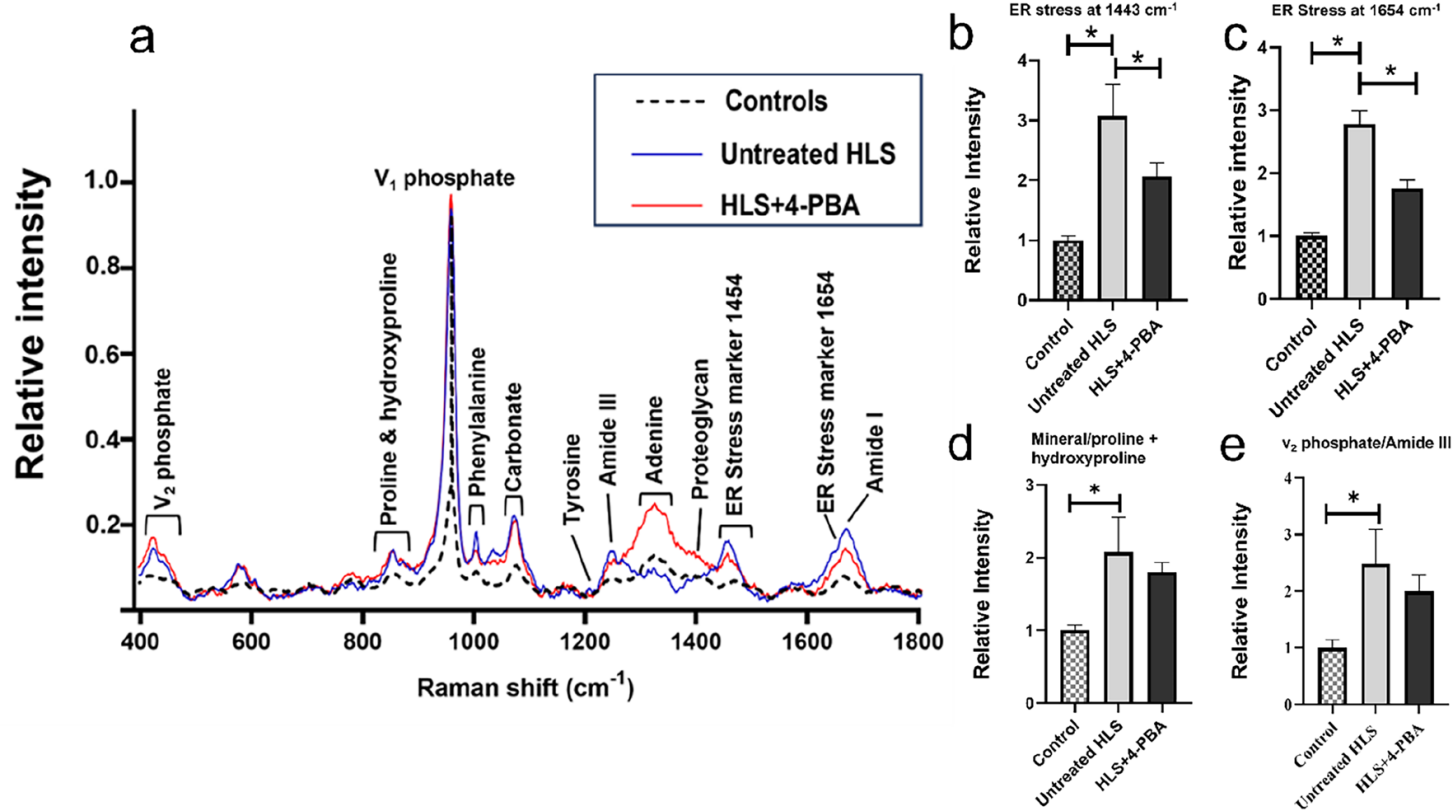
Baseline-corrected Raman spectra analysis of cortical bones, specific ER stress marker bands, and mineral-to-matrix ratio quantification measured by Raman spectroscopy among the control, untreated HLS, and HLS + 4-PBA treated groups. (**a**) Superimposed Raman spectra analysis of cortical bone of three study groups. The X-axis shows different peaks described by wave number labeled in cm^−1^, while the Y-axis displays the relative intensity of peaks. Labeled peaks depict major bone mineral and matrix collagen band positions to compare the differences between untreated HLS and HLS + 4-PBA groups in comparison to the control group. (**b**) Raman Bands at 1443 cm^−1^, (**c**) at 1654 cm^−1^ band, (**d**) mineral/proline + hydroxyproline at (960 cm^−1^/922 + 855 + 876 cm^−1^), and (e) v_2_ phosphate/amide III at (430 cm^−1^/1242 cm^−1^) identified in the three study groups (*p < 0.05). Data are expressed as mean ± SEM. (n = 5/group) (HLS: hindlimb suspension; 4-PBA: 4- phenylbutyrate).

Major peaks related to the mineral part of bone was quantitatively measured, and the most intense band represents the *v*_1_ mode of the phosphate ion (v_1_PO_4_^3−^) in hydroxyapatite at about (960 cm^−1^) detected among all the three study groups. A prominent carbonate band (v_1_CO_3_^2−^) around (1070 cm^−1^) can be identified as well in all three groups, and a phosphate peak of (v_2_PO_4_^3−^) was observed at (430 cm^−1^). Organic matrix contents, in particular, amide I at (1660 cm^−1^ and 1690 cm^−1^), amide III at (1242 cm^−1^), and amino acids residues and proteins – phenylalanine at (1003 cm^−1^), proline (855 cm^−1^), hydroxyproline at (876 cm^−1^), CH_2_ Wag at (1450 cm^−1^), tyrosine at (1204 cm^−1^), proteoglycan at (1375 cm^−1^) and adenine (1325 cm^−1^) were shown and labeled in the graph (Fig. 3a).

Specific ER stress markers at bands (1443 cm^−1^) and (1654 cm^−1^) were examined (Fig. 3a) and compared among the three study groups (Fig. 3b and c). Spectra at bands (1443 cm^−1^) (Fig. 3b) and (1654 cm^−1^) (Fig. 3c) were detected in untreated HLS group and demonstrated a statistically significant increase in intensity (*p* < 0.05) when compared to the control group. Upon treatment with 4-PBA; the levels of ER stress in the HLS + 4-PBA group were lower than in the untreated HLS group (*p* < 0.05; Fig. 3b and c). Our results showed that mineral-to-matrix ratio at mineral/proline + hydroxyproline (960 cm^−1^/922 + 855 + 876 cm^−1^) and (v_2_PO_4_^3^/Amide III) at (430 cm^−1^/1242 cm^−1^) were significantly higher in untreated HLS group compared to the control group and the ratio tend to be lower in HLS + 4-PBA group (*p* < 0.05; Fig. 3d and e).

**Figure 4:**
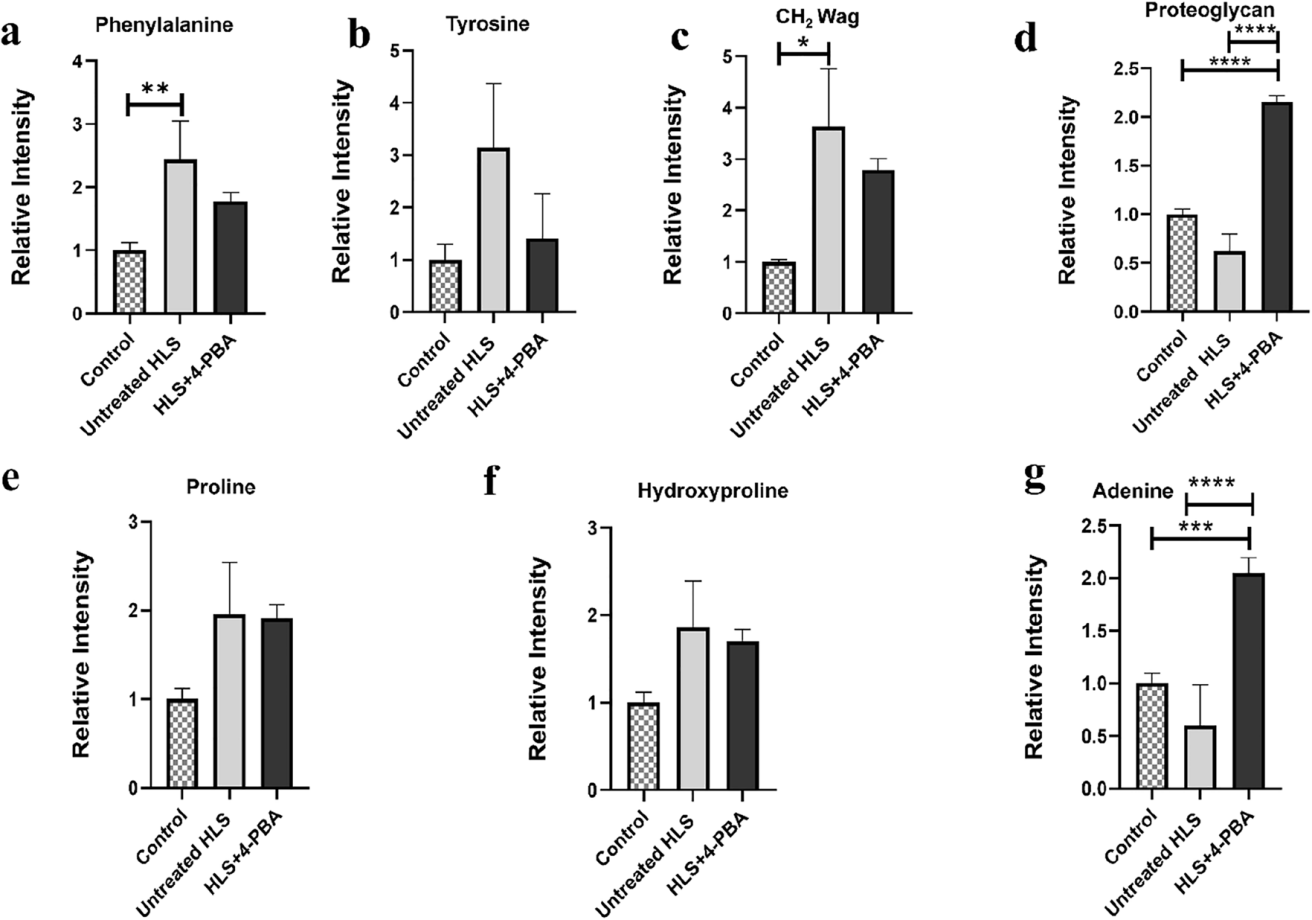
Raman peak measurements for amino acids and bone-related proteins among control, untreated HLS, and HLS + 4-PBA groups (**a**) Phenylalanine at (1003 cm^−1^), (**b**) Tyrosine at (1204 cm^−1^), (**c**) CH_2_ Wag at (1450 cm^−1^), (**d**) Proteoglycan at (1375 cm^−1^), (**e**) proline ratio at (855 cm^−1^), (**f**) hydroxyproline at (876 cm^−1^), and (**g**) Adenine at (1325 cm^−1^). (*p < 0.5, **p < 0.01, ***p < 0.001, ****p < 0.0001). Data are expressed as mean ± SEM. (n = 5/group) (HLS: hindlimb suspension; 4-PBA: 4- phenylbutyrate).

The Raman peak intensity ratios were calculated and compared amongst the three study groups to quantitatively validate spectrum alterations associated with amino acids and bone-related proteins, as illustrated in (Fig. 4). Phenylalanine at (1003 cm^−1^) band was higher in the untreated HLS group than the control group (*p* < 0.01; Fig. 4a). Tyrosine at (1204 cm^−1^) was also higher in the untreated HLS group and lower in the HLS + 4-PBA group (Fig. 4b). CH_2_-Wag at (1450 cm^−1^) was significantly higher in the untreated HLS group than the control group (*p* < 0.5; Fig. 4c). Proteoglycan levels at the band (1375 cm^−1^) were found to be significantly lower (*p* < 0.0001) in untreated HLS but higher in HLS + 4-PBA treated group (*p* < 0.0001; Fig. 4d). Proline at (855 cm^−1^) was higher in the untreated HLS group than in the control group (*p* < 0.5; Fig. 4e). Hydroxyproline at (876 cm^−1^) had a similar trend to proline at (855 cm^−1^) (Fig. 4f). Adenine, a nucleic acid, at (1325cm^−1^), was found to be significantly higher in the HLS + 4-PBA group (*p* < 0.0001; Fig. 4g).

**Figure 5:**
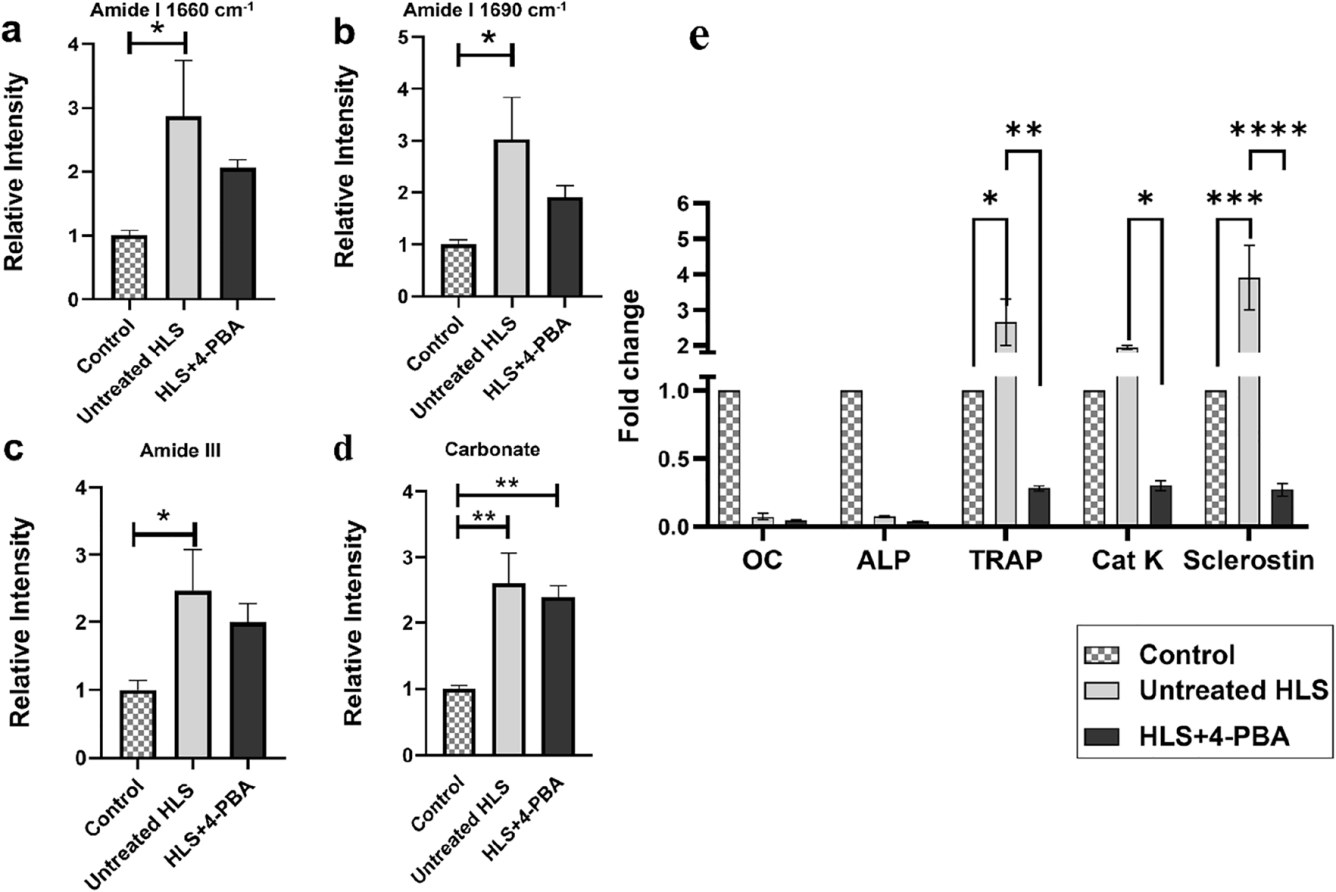
Raman peak assignment for collagen crosslinking for amide I, amide III, carbonate ratios, and relative mRNA levels of bone turnover markers among control, untreated HLS, and HLS + 4-PBA groups. (**a**) amide I band at (1660 cm^−1^), (**b**) amide I band at (1690 cm^−1^), (**c**) amide III band at (1242 cm^−1^), (**d**) carbonate at (1070 cm^−1^). (**e**) The expression levels of bone formation markers for osteoblasts, OC, and ALP, bone resorption for osteoclasts, TRAP, and Cat K, and osteocytes marker sclerostin. Data are expressed as mean ± SEM. (n = 5/group);(n = 3/group). (HLS; hindlimb suspension, 4-PBA; 4-phenyl butyrate, OC; osteocalcin, ALP; alkaline phosphatase, TRAP; Tartrate-Resistant Acid Phosphatase, Cat K; Cathepsin K) (*p < 0.05, **p < 0.01, ***p < 0.001; ****p < 0.0001).

In untreated HLS group, amide I was detected at (1660 cm^−1^) and at (1690 cm^−1^) bands and were significantly higher than the control (*p* < 0.05; Fig. 5a and b). The Amide III band at (1242 cm^−1^) was higher in the untreated HLS group and lower with 4-PBA treatment (Fig. 5c). To quantitatively validate spectral changes among the study groups related to collagen crosslink maturity ratio (1660/1690 cm^−1^), the Raman peak intensity ratios were calculated and compared between the three groups, as shown in (supplementary Fig. 5a). There is a reduction in the ratio of amide I (1660/1690 cm^−1^) in untreated HLS, while the ratio was comparatively higher in HLS + 4-PBA treated group.

Carbonate content was compared, and the untreated HLS group showed significantly higher levels of carbonate at the (1070 cm^−1^) band (*p* < 0.01) (Fig. 5d), while treatment with 4-PBA in HLS + 4-PBA group showed a reduction of carbonate but still significantly higher than the control group. The carbonate-to-phosphate ratio was compared to assess mineral quality among the groups. The carbonate-to-phosphate ratio at (1070 cm^−1^/430 cm^−1^) in untreated HLS was higher than that in the HLS + 4-PBA group (*p* < 0.05; supplementary Fig. 5b).

### 4-PBA reduced the mRNA expressions of bone resorption markers

There was no difference observed among the study groups concerning mRNA levels of bone formation markers ALP and OC (Fig. 5e). The mRNA levels of bone resorption markers (TRAP) and cathepsin K (Cat K) were higher in the untreated HLS group compared to the control group. However, upon treatment with 4-PBA, the HLS + 4-PBA group showed downregulation of TRAP and Cat K compared to the untreated HLS (*p* < 0.01; *p* < 0.05; Fig. 5e). The mRNA levels of sclerostin were significantly higher in untreated HLS compared to the control group, (*p* < 0.001; Fig. 5e), but the level reduced significantly in HLS + 4-PBA treated group (*p* < 0.0001; Fig. 5e).

## Discussion

Disuse osteoporosis frequently occurs in bedridden patients as well as among astronauts returning from space to Earth, nevertheless, the precise mechanisms underlying this condition remain elusive. To resolve its etiology and explore potential therapeutic interventions, numerous studies have utilized animal models that simulate disuse osteoporosis.

In this study, we developed a model of disuse osteoporosis using adult 4-month-old C57BL/6J mice using an established hindlimb suspension technique and investigated the therapeutic intervention with 4-PBA^14^. The mice had attained skeletal maturity by 4 months, allowing us to evaluate bone changes in a stable adult skeleton rather than in a growing skeleton^36^.

While the pharmacological treatment of bone loss is still in its early stages, the current study showed evidence that alleviating ER stress and bone resorption markers secondary to hindlimb suspension with daily 4-PBA treatment for three weeks mitigates disuse osteoporosis in the hindlimb bones in mice, as observed by histology and micro-CT and supported by physicochemical characteristics using Raman spectroscopy and gene expression analysis.

This study found that three weeks of hindlimb suspension in mice resulted in the generation of ROS and oxidative stress, as well as a 10% decrease in body weight. Although all animals remained generally active and healthy, oxidative stress in the skeletal organ may have a negative impact on mesenchymal stem cells (MSCs) contribution to the bone remodeling process, leading to an increase in bone resorption and decrease in bone formation, reducing bone mass and bone mineral density and establishing the development of osteoporosis^37^. Previous studies have examined the interaction between oxidative stress, the ER stress response, inflammation, immunity, and the resultant metabolic disease^38^.

HLS during this study caused cortico-cancellous morphological changes in the untreated HLS group, demonstrating a reduced number and thickness of trabecular structures and their interconnectivity, a decrease in the number of osteocytes, and an increased number of adipocytes in the hindlimb marrow. These findings were further supported by changes in the microarchitecture of the bones in micro-CT. In addition, gene expression study revealed increases in the osteoclast markers TRAP and Cat K, upregulation of sclerostin levels by osteocytes, and lower expression of osteoblast markers ALP and OC. Further investigations denote the presence of ER stress in the untreated HLS group, as indicated by Raman spectroscopy. This unresolved ER stress is the cause of the imbalance in bone remodeling noticeable in the untreated HLS group.

In the current study, HLS mice treated with daily 4-PBA administration for 21 days demonstrated a significant degree of restoration of hindlimb trabecular morphology. This restoration was associated with a notable increase in osteocyte cell density and concurrent reduction in the abundance of adipocytes in the hindlimb marrow. Moreover, micro-CT analysis revealed that 4-PBA treatment substantially improved trabecular microarchitecture and enhanced cortical structure resulting in enhanced bone volume parameters. Furthermore, Raman spectroscopy indicated a decline in ER stress markers at 1443 cm^−1^ and 1654 cm^−1^ bands in HLS + 4-PBA-treated group. These findings were supported by gene expression results that demonstrated a reduction in ER stress resulting in downregulated TRAP and Cat K expression in osteoclasts alongside reduced sclerostin expression in osteocytes; consequently, reducing osteoclastogenesis and promoting osteoblastogenesis.

Treatment with 4-PBA also demonstrated enhancement of physicochemical properties of the hindlimb-suspended bones extracellular matrix following Raman spectroscopic analysis. Our findings suggest that 4-PBA ameliorated ER stress-induced bone loss in the murine hindlimb suspension model. 4-PBA is a chemical chaperone, that acts as an ammonia scavenger and has FDA approval for treatment of urea cycle disorders.

The high expressions of sclerostin observed in this study were plausibly driven by the absence of mechanical stimulation on osteocytes that generated ER stress and oxidative stress, which stimulated RANKL production and promoted osteoclastogenesis.

Another finding in our histological evaluation was a significantly high number of adipocytes in the bone marrow of the untreated HLS group, which was consistent with the finding of Zou et al.^39^. Bone marrow adipocytes (BMAs) are distinct from peripheral adipose tissue because they originate from bone marrow mesenchymal stromal cells (BMSCs) which is similar to osteoblast lineage^40^. BMAs act in a paracrine mode on bone cells, influencing osteoclasts via its RANKL secretion^40^ and regulating osteoblasts by secretion of Wnt inhibitors^41^. Consequently, they enhance osteoclastogenesis and reduce osteoblastogenesis. Due to their common lineage with osteoblasts, BMAs compete with them by favoring BMSCs differentiation into adipocytes rather than osteoblasts^42^ under oxidative stress and proinflammatory conditions^43^. In this study, we discovered that when the hindlimb-suspended group were treated with 4-PBA, the number of dense adipocytes decreased while the number of osteocytes increased. Basseri et al.have also shown that ER stress activation contributed to adipogenesis and blocking it with 4-PBA prevented adipocyte differentiation^19^, corroborating with our findings.

In the micro-CT analysis, loss of bone was evident in the untreated HLS group with significant changes in the trabecular and cortical bone compartments similar to a recent finding by Steczina et al.^28^, while 4-PBA treatment restored the reduced bone volume fraction, the reduction of trabecular thickness in the untreated HLS group is primarily caused by the conversion of trabeculae plates into rod structures, possibly due to perforation of the trabecular plates by the action of osteoclasts; and leading to complete removal of trabeculae in many sites resulting in loss of trabecular interconnectivity^44^. The variance in trabecular bone mechanical characteristics in untreated HLS group was demonstrated by a decrease in trabecular anisotropy and bone volume fraction as shown in micro-CT analysis of the tibia. This loss of trabeculae together with a reduction in anisotropy, may affect the mechanical properties of the bone, producing the risk of fragility fracture^45^.

In the present study, ER stress was found to be involved in defective osteoblasts functions as observed in Raman parameters of mineral-to-matrix ratio and the collagen maturity quality whereas suppression of ER stress by 4-PBA treatment resulted in favorable enhancement of mineral-to-matrix ratio, enhanced collagen maturity, and cross-linking levels and amino acids and proteins found in the extracellular matrix composition.

The decrease in mature matrix crosslinks in this study as measured by the ratio of amide I (1660 cm^−1^) over amide I (1690 cm^−1^), may be due to increased collagen (Col1) breakdown and decreased mineralization of new collagen due to suppression of osteoblastogenesis observed in disuse conditions as demonstrated by Buettmann et al.^46^.

In this study, the carbonate levels in untreated HLS were found to be higher, signifying an increase in bone brittleness, and that it corresponds to increased fragility of the hindlimbs. As with 4-PBA treatment, hindlimbs carbonate substitution showed a trend towards reduction. The increase of phenylalanine levels in untreated HLS indicates an increase in bone resorption due to disuse, whereas the 4-PBA-treated group showed a downward trend. CH_2_ Wag (1450 cm^−1^) Raman band represents the amount of all organic matrix components including collagen, lipids, and non-collagenous proteins, and is regarded as a general marker of protein content. It was found altered in untreated HLS group and it could be reversed with 4-PBA treatment. Tyrosine levels in the HLS + 4-PBA group were reduced compared to the control group, hence suggesting a reduction in osteoclast activity and differentiation^47,48^. The untreated HLS group has fewer proteoglycans, which is associated with reduced bone strength, as seen clinically in bone fragility due to disuse osteoporosis. Meanwhile, the HLS + 4-PBA treatment group has significantly higher levels of proteoglycans, which may contribute to more favorable osteoblasts’ function, potentially improving bone strength in the 4-PBA treatment group.

Adenine, a purine, is involved in protein synthesis and cellular respiration. In our study, adenine levels were significantly higher in the 4-PBA treatment group. Chen et al.^49^ discovered that adenine exerts anti-inflammatory activity and promotes osteoblast differentiation in normal and inflammatory experimental conditions. In the current study, 4-PBA treatment significantly increased adenine levels, promoting an anti-inflammatory effect in the 4-PBA treatment group.

Raman spectroscopic analysis of physicochemical bone properties in untreated HLS group revealed a poorer ability to support high-quality mineralization, and less collagen crosslinking, whereas treatment with 4-PBA in HLS + 4-PBA group showed an increase in mineralization and more collagen crosslinking.

In this study, we found that ALP and OC were downregulated in both untreated HLS and HLS + 4-PBA groups, suggesting poorer differentiation capacity of osteoblast in both groups. ALP expression levels were found to be downregulated in other microgravity simulation and hindlimb suspension studies when compared to the control group^50–54^. Since there was a high number of adipocytes in the histological evaluation in this study, our findings suggest that MSCs have a predilection of fat cell lineage differentiation and a reduced choice preference for pre-osteoblast differentiation, which explains the downregulation of ALP expression.

OC gene expression was high in the control group but decreased abruptly in untreated HLS group and remained low despite 4-PBA treatment among HLS + 4-PBA group. OC is a protein secreted by mature osteoblasts, that is responsible for matrix mineralization of newly formed bone^55^. Other studies have shown that hindlimb suspension is also associated with lower mRNA levels of OC^56^.

The inability of 4-PBA treatment to upregulate osteocalcin expressions in this study was most likely due to the poorer quality of ECM produced as a result of ER stress which resulted in an unfavorable mineralization process. In addition, other studies have demonstrated morphological changes in osteoblasts under simulated microgravity conditions due to actin cytoskeleton disruptions producing spheroidal osteoblasts^57,58^. We hypothesize that increasing osteocalcin levels after 4-PBA treatment in this study could be achieved by administering 4-PBA treatment for a longer duration beyond 21 days or delivered at a higher therapeutic dose.

Investigation on bone resorption markers in this study found that both TRAP and Cat K were upregulated in the untreated HLS group. In this study, therapeutic treatment with 4-PBA downregulated both TRAP and Cat K simultaneously. In other studies, treatment with 4-PBA downregulated Cat K only, but did not affect the mRNA level of TRAP. This disparity in results could be attributed to different osteoclast physiological functions in various skeleton sites^59,60^.

The density of osteocytes in the untreated HLS group is significantly lower than the control group in this study, whereas treatment with 4-PBA in HLS + 4-PBA significantly increased the number of osteocytes compared to untreated HLS group, but the density is still lower than the control. This finding is correlated with the persistently low levels of expressions of ALP and OC despite 4-PBA treatment in HLS + 4-PBA group.

Besides observing osteoblastogenic and osteoclastogenic gene expressions, we have also measured sclerostin released by osteocytes. Sclerostin inhibits bone formation by inhibiting the Wnt/β catenin signaling which is responsible for osteoblasts differentiation and proliferation. Expression of sclerostin is regulated by mechanical unloading in osteocytes^61^. The reduction in osteocyte cellular density in the untreated HLS group in this study could be due to osteocyte apoptosis caused by disuse conditions that commensurate with the high amount of sclerostin expression which led to osteoclastogenesis. Uncontrolled ER stress caused by hindlimb suspension for 21 days may have exacerbated osteocyte apoptosis. Liu et al.investigated the effects of ER stress activation in simulated microgravity that lead to changes in mechano-sensation and apoptosis in osteocytes, and they were able to reduce these effects with an ER stress inhibitor melatonin^62^ whereas we were able to reverse the ER stress and downregulated sclerostin expressions using 4-PBA.

Collectively, our findings, support the role of ER stress as a causative factor in disuse-induced bone loss, and mitigating ER stress with 4-PBA is a novel approach to restore bone homeostasis in vivo. Disuse stress on bone cells osteoblasts, osteoclasts, and osteocytes caused by HLS leads to malformation of both mineral and organic content of the extracellular matrix (ECM).

This study suggests that ER stress-induced cellular effects can be identified as changes in the Raman spectra^31^. While treatment with 4-PBA in HLS + 4-PBA group showed a trend towards ER stress reduction, the level attained was insignificant. This result may be due to the lower dose of 4-PBA administered in our study (100mg/kg/d) compared to Park et al. who obtained a favorable result after using a much higher dose of 4-PBA at 240 mg/kg/d for their lipopolysaccharide (LPS)-osteoporosis model^59^. On the contrary, Yamada et al.also achieved a favorable result when using a much lower 4-PBA dose of 24mg/kg/d as compared to this study, in their periodontitis model induced by *P gingivalis*^63^. These variations in results highlighted the differences in responses to 4-PBA treatment in the management of ER stress in diverse pathological conditions, which deserve further investigations.

## Conclusion

We concluded that elevated ER stress is involved in the pathogenesis of osteoporosis due to mechanical unloading in HLS mice. The use of 4-PBA treatment establishes a mechanistic link between elevated ER stress and disuse-induced osteoporosis by partially restoring the morphology, physicochemical characteristics, and gene expression profile of hindlimbs in HLS mice. Furthermore, 4-PBA appears to demonstrate a robust translational potential for osteoporosis prevention. Further studies are warranted to thoroughly characterize the therapeutic potential of 4-PBA before attempting experimental clinical interventions.

### Supplementary Information

Supplementary Information.

## Data Availability

All the gene expression data is deposited in ArrayExpress with accession E-MTAB-13529.

## References

[1] Hernlund E (2013). Osteoporosis in the European Union: medical management, epidemiology, and economic burden. Arch. Osteoporos..

[2] Cooper C (2011). Secular trends in the incidence of hip and other osteoporotic fractures. Osteoporos. Int..

[3] Hadjidakis DJ, Androulakis II (2006). Bone remodeling. Ann. N. Y. Acad. Sci..

[4] Walter P, Ron D (2011). The unfolded protein response: From stress pathway to homeostatic regulation. Science (80-)..

[5] Bohnert KR, McMillan JD, Kumar A (2018). Emerging roles of ER stress and unfolded protein response pathways in skeletal muscle health and disease. J. Cell. Physiol..

[6] Yoshida H (2007). ER stress and diseases. FEBS J..

[7] Hetz C (2012). The unfolded protein response: controlling cell fate decisions under ER stress and beyond. Nat. Rev. Mol. cell Biol..

[8] Chen, H. Review on the endoplasmic reticulum stress and related diseases. in *2021 3rd International Conference on Intelligent Medicine and Image Processing* 163–168 (2021).

[9] Malhotra JD, Kaufman RJ (2007). Endoplasmic reticulum stress and oxidative stress: A vicious cycle or a double-edged sword?. Antioxidants Redox Signal..

[10] Jäger R, Bertrand MJM, Gorman AM, Vandenabeele P, Samali A (2012). The unfolded protein response at the crossroads of cellular life and death during endoplasmic reticulum stress. Biol. Cell.

[11] Komori T (2015). Animal models for osteoporosis. Eur. J. Pharmacol..

[12] Sato AY, Tu X, McAndrews KA, Plotkin LI, Bellido T (2015). Prevention of glucocorticoid induced-apoptosis of osteoblasts and osteocytes by protecting against endoplasmic reticulum (ER) stress in vitro and in vivo in female mice. Bone.

[13] Liu L, Zhang Y, Gu H, Zhang K, Ma L (2015). Fluorosis induces endoplasmic reticulum stress and apoptosis in osteoblasts in vivo. Biol. Trace Elem. Res..

[14] Morey ER (1979). Spaceflight and bone turnover: Correlation with a new rat model of weightlessness. Bioscience.

[15] Komori T (2015). Animal models for osteoporosis. Eur. J. Pharmacol..

[16] Morey-Holton E, Globus RK, Kaplansky A, Durnova G (2005). The hindlimb unloading rat model: Literature overview, technique update and comparison with space flight data. Adv. Space Biol. Med..

[17] Zhong M, Wu Z, Chen Z, Ren Q, Zhou J (2023). Advances in the interaction between endoplasmic reticulum stress and osteoporosis. Biomed. Pharmacother..

[18] Duran, I. *et al.* 4-PBA Treatment improves bone phenotypes in the mouse model of osteogenesis imperfecta. *J. Bone Miner. Res. Off. J. Am. Soc. Bone Miner. Res.* **37**, 675–686 (2022). 10.1002/jbmr.4501 PMC9018561 34997935

[19] Basseri S, Lhoták Š, Sharma AM, Austin RC (2009). The chemical chaperone 4-phenylbutyrate inhibits adipogenesis by modulating the unfolded protein response. J. Lipid Res..

[20] Yang J, Wu Q, Lv J, Nie H (2017). 4-Phenyl butyric acid prevents glucocorticoid-induced osteoblast apoptosis by attenuating endoplasmic reticulum stress. J. Bone Miner. Metab..

[21] Khan, A. A. *et al.* Mitigating sarcoplasmic reticulum stress limits disuse-induced muscle loss in hindlimb unloaded mice. *npj Microgravity* **8**, 24 (2022). 10.1038/s41526-022-00211-w PMC9273600 35817772

[22] Kilkenny C, Browne WJ, Cuthill IC, Emerson M, Altman DG (2010). Improving bioscience research reporting: The ARRIVE guidelines for reporting animal research. PLOS Biol..

[23] Cao A-L (2016). Ursodeoxycholic acid and 4-phenylbutyrate prevent endoplasmic reticulum stress-induced podocyte apoptosis in diabetic nephropathy. Lab. Investig..

[24] Brent, M. B., Brüel, A. & Thomsen, J. S. A systematic review of animal models of disuse-induced bone loss. *Calcif. Tissue Int.* 1–15 (2021). 10.1007/s00223-020-00799-9 33386477

[25] Dempster, D. W. *et al.* Standardized nomenclature, symbols, and units for bone histomorphometry: a 2012 update of the report of the ASBMR Histomorphometry Nomenclature Committee. *J. bone Miner. Res. Off. J. Am. Soc. Bone Miner. Res.* **28**, 2–17 (2013). 10.1002/jbmr.1805 PMC3672237 23197339

[26] Bouxsein ML (2010). Guidelines for assessment of bone microstructure in rodents using micro–computed tomography. J. Bone Miner. Res..

[27] Ellman R (2014). Combined effects of botulinum toxin injection and hind limb unloading on bone and muscle. Calcif. Tissue Int..

[28] Steczina S (2020). Dietary countermeasure mitigates simulated spaceflight-induced osteopenia in mice. Sci. Rep..

[29] Klintström E, Smedby Ö, Moreno R, Brismar TB (2014). Trabecular bone structure parameters from 3D image processing of clinical multi-slice and cone-beam computed tomography data. Skeletal Radiol..

[30] Karim A (2022). Hindlimb unloading induces time-dependent disruption of testicular histology in mice. Sci. Rep..

[31] Hosoda A (2011). Detection of ER stress in vivo by Raman spectroscopy. Biochem. Biophys. Res. Commun..

[32] Wang H (2011). Depth-resolved in vivo micro-Raman spectroscopy of a murine skin tumor model reveals cancer-specific spectral biomarkers. J. Raman Spectrosc..

[33] López-Díez EC, Winder CL, Ashton L, Currie F, Goodacre R (2005). Monitoring the mode of action of antibiotics using Raman spectroscopy: Investigating subinhibitory effects of amikacin on pseudomonas aeruginosa. Anal. Chem..

[34] Chen X (2019). Surface-enhanced Raman scattering method for the identification of methicillin-resistant Staphylococcus aureus using positively charged silver nanoparticles. Microchim. Acta.

[35] Ishimaru Y (2018). Raman spectroscopic analysis to detect reduced bone quality after sciatic neurectomy in mice. Molecules.

[36] Beamer WG, Donahue LR, Rosen CJ, Baylink DJ (1996). Genetic variability in adult bone density among inbred strains of mice. Bone.

[37] Zhang C (2023). Oxidative stress: A common pathological state in a high-risk population for osteoporosis. Biomed. Pharmacother..

[38] Dandekar A, Mendez R, Zhang K (2015). Cross talk between ER stress, oxidative stress, and inflammation in health and disease. Methods Mol. Biol..

[39] Zou, Y. *et al.* Evaluation of microalgae on preventing bone loss in C57BL/6J mice induced by hindlimb suspension. *Food Front.* (2023).

[40] Muruganandan S, Sinal CJ (2014). The impact of bone marrow adipocytes on osteoblast and osteoclast differentiation. IUBMB Life.

[41] Taipaleenmäki H, Abdallah BM, AlDahmash A, Säämänen A-M, Kassem M (2011). Wnt signalling mediates the cross-talk between bone marrow derived pre-adipocytic and pre-osteoblastic cell populations. Exp. Cell Res..

[42] Jafari A (2017). Legumain regulates differentiation fate of human bone marrow stromal cells and is altered in postmenopausal osteoporosis. Stem Cell Rep..

[43] Lin C-H, Li N-T, Cheng H-S, Yen M-L (2018). Oxidative stress induces imbalance of adipogenic/osteoblastic lineage commitment in mesenchymal stem cells through decreasing SIRT1 functions. J. Cell. Mol. Med..

[44] Felder AA (2021). The plate-to-rod transition in trabecular bone loss is elusive. R. Soc. Open Sci..

[45] Ishijima, M. *et al.* Resistance to unloading-induced three-dimensional bone loss in osteopontin-deficient mice. *J. Bone Miner. Res. Off. J. Am. Soc. Bone Miner. Res.* **17**, 661–667 (2002). 10.1359/jbmr.2002.17.4.661 11918223

[46] Buettmann EG (2022). Similarities between disuse and age-induced bone loss. J. Bone Miner. Res..

[47] Shalev M, Elson A (2019). The roles of protein tyrosine phosphatases in bone-resorbing osteoclasts. Biochim. Biophys. acta. Mol. cell Res..

[48] Pallu S (2012). Synchrotron ultraviolet microspectroscopy on rat cortical bone: involvement of tyrosine and tryptophan in the osteocyte and its environment. PLoS One.

[49] Chen, Y.-P. *et al.* Anti-inflammatory effects of adenine enhance osteogenesis in the osteoblast-like MG-63 Cells. *Life* vol. 10 (2020). 10.3390/life10070116 PMC7399991 32707735

[50] He J-P (2018). Icariin prevents bone loss by inhibiting bone resorption and stabilizing bone biological apatite in a hindlimb suspension rodent model. Acta Pharmacol. Sin..

[51] Bucaro MA (2007). The effect of simulated microgravity on osteoblasts is independent of the induction of apoptosis. J. Cell. Biochem..

[52] Li J, Geng J, Lin T, Cai M, Sun Y (2022). A mouse model of disuse osteoporosis based on a movable noninvasive 3D-printed unloading device. J. Orthop. Transl..

[53] Kostenuik PJ, Halloran BP, Morey-Holton ER, Bikle DD (1997). Skeletal unloading inhibits the in vitro proliferation and differentiation of rat osteoprogenitor cells. Am. J. Physiol..

[54] Rucci, N. *et al.* Lipocalin 2: a new mechanoresponding gene regulating bone homeostasis. *J. bone Miner. Res. Off. J. Am. Soc. Bone Miner. Res.* **30**, 357–368 (2015). 10.1002/jbmr.2341 25112732

[55] Pockwinse SM, Wilming LG, Conlon DM, Stein GS, Lian JB (1992). Expression of cell growth and bone specific genes at single cell resolution during development of bone tissue-like organization in primary osteoblast cultures. J. Cell. Biochem..

[56] Shuang F (2013). Destrin deletion enhances the bone loss in hindlimb suspended mice. Eur. J. Appl. Physiol..

[57] Gioia M (2018). Simulated microgravity induces a cellular regression of the mature phenotype in human primary osteoblasts. Cell Death Discov..

[58] Mann, V. *et al.* Changes in human foetal osteoblasts exposed to the random positioning machine and bone construct tissue engineering. *Int. J. Mol. Sci.* **20**, (2019). 10.3390/ijms20061357 PMC6471706 30889841

[59] Park H-J, Son H-J, Sul O-J, Suh J-H, Choi H-S (2018). 4-Phenylbutyric acid protects against lipopolysaccharide-induced bone loss by modulating autophagy in osteoclasts. Biochem. Pharmacol..

[60] Everts V, de Vries TJ, Helfrich MH (2009). Osteoclast heterogeneity: Lessons from osteopetrosis and inflammatory conditions. Biochim. Biophys. Acta Mol. Basis Dis..

[61] Suen PK, Qin L (2016). Sclerostin, an emerging therapeutic target for treating osteoporosis and osteoporotic fracture: A general review. J. Orthop. Transl..

[62] Liu L-J, Li S, Wu X-T, Yang X, Sun L-W (2022). Contribution of endoplasmic reticulum stress response to the mechanosensitivity alteration in osteocytes under simulated microgravity. Acta Astronaut..

[63] Yamada H, Nakajima T, Domon H, Honda T, Yamazaki K (2015). Endoplasmic reticulum stress response and bone loss in experimental periodontitis in mice. J. Periodontal Res..

