# Supplementary material for: Pharmacological inhibition of endoplasmic reticulum stress mitigates osteoporosis in a mouse model of hindlimb suspension
Source: Sci Rep. 2024 Feb 27;14:4719. doi: 10.1038/s41598-024-54944-7 (PMC10899598; doi:10.1038/s41598-024-54944-7)
Supplement: Supplementary file 1 — Supplementary Information. [file 41598_2024_54944_MOESM1_ESM.docx]

**pHARMACOLOGICAL INHIBITION OF ENDOPLASMIC RETICULUM STRESS MITIGATES OSTEOPOROSIS IN A MOUSE MODEL OF HINDLIMB SUSPENSION**

**Hiba Al-Daghestani^1^, Rizwan Qaisar^2,3^, Sausan Al Kawas^1^, Nurhafizah Ghani^4^, Aghila Rani K.G. ^5^, Muhammad Azeem^6^, Hijaz Kamal Hasnan^7^, Nur Karyatee Kassim^4*^ and A.R. Samsudin^1*^**

^1^Department of Oral and Craniofacial Health Sciences, College of Dental Medicine, University of Sharjah, Sharjah 27272, UAE

^2^Department of Basic Medical Sciences, College of Medicine, University of Sharjah, Sharjah 27272, UAE

^3^Space Medicine Research Group, Research Institute for Medical and Health Sciences, University of Sharjah, Sharjah 27272, UAE

^4^School of Dental Sciences, Health Campus, Universiti Sains Malaysia, Kubang Kerian 16150, Kelantan, Malaysia

^5^Research Institute for Medical and Health Sciences, University of Sharjah, Sharjah 27272, UAE

^6^Department of Mathematical and Physical Sciences, University of Nizwa, Nizwa 33, Sultanate of Oman

**^7^**Department of Geology, Faculty of Science, University of Malaya, Kuala Lumpur, Malaysia

**^*^ Correspondence**: [drabrani@sharjah.ac.ae](mailto:drabrani@sharjah.ac.ae); karyatee@usm.my

# Supplementary figures


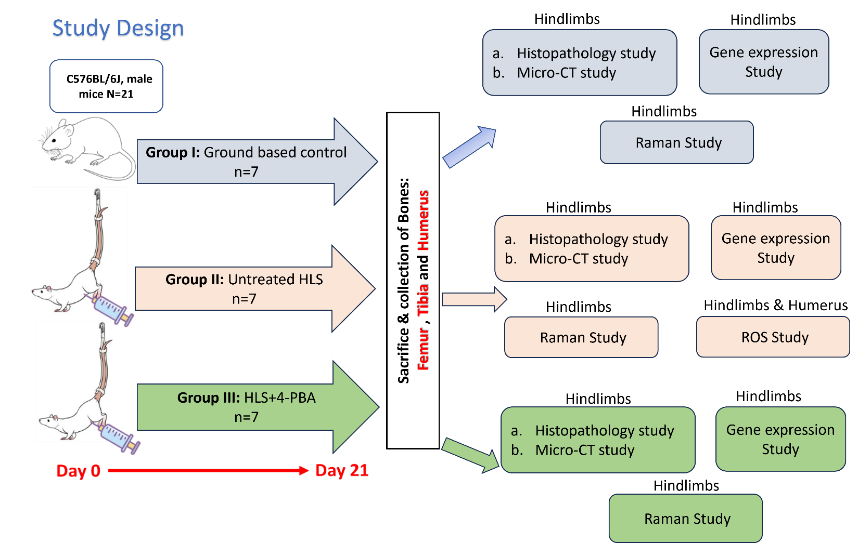


**Supplementary Fig 1. Flow Chart of Study.**

(HLS; hindlimb suspension, ROS; reactive oxygen species, 4-PBA; 4-phenyl butyrate).


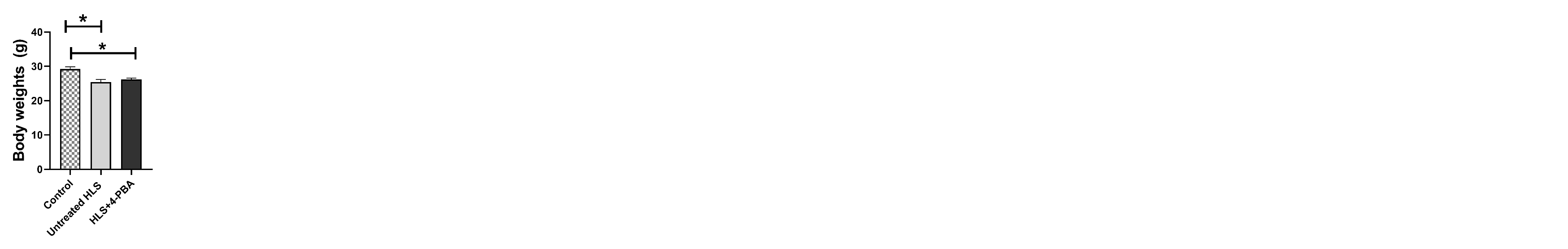


**Supplementary Fig 2. Bodyweight (g) of experimental groups, among control, untreated HLS, and HLS+4-PBA treated mice**. (a) Bodyweight (g) of experimental groups where both untreated HLS and HLS+4-PBA groups suffered a significant loss in body weight compared to control, while HLS+4-PBA mice that received treatment with 4-PBA demonstrated a slightly higher body weight compared to untreated HLS. Data are expressed as mean ± SEM. (*p<0.05)

**
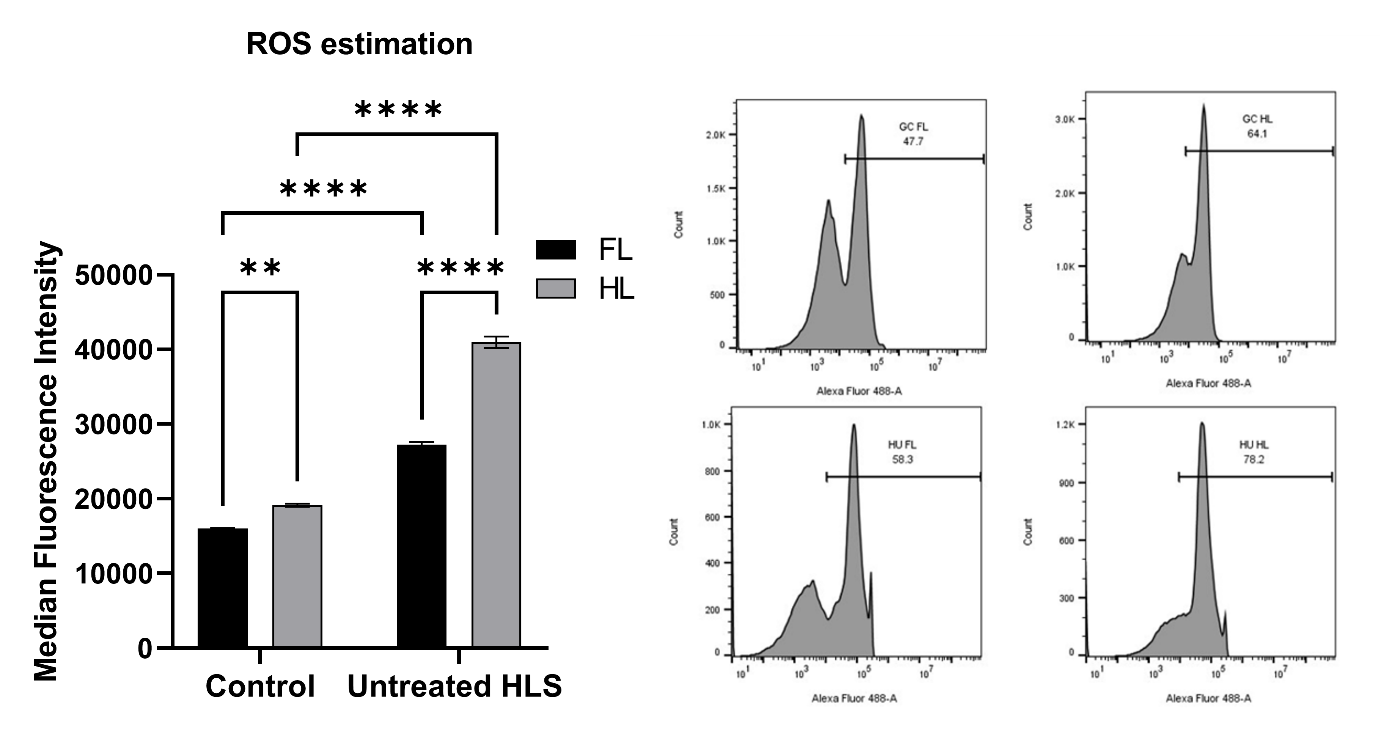
**

**Supplementary Fig 3. ROS measurement in untreated HLS compared to control**. ROS levels in hindlimbs of untreated HLS group were significantly higher than forelimbs (p<0.0001), while controls showed a much lower level of ROS. Data are expressed as mean ± SEM. (ROS; reactive oxygen species, HLS; hindlimb suspension, HL; Hindlimbs, FL; forelimbs). (****p< 0.0001; **p<0.01).

**
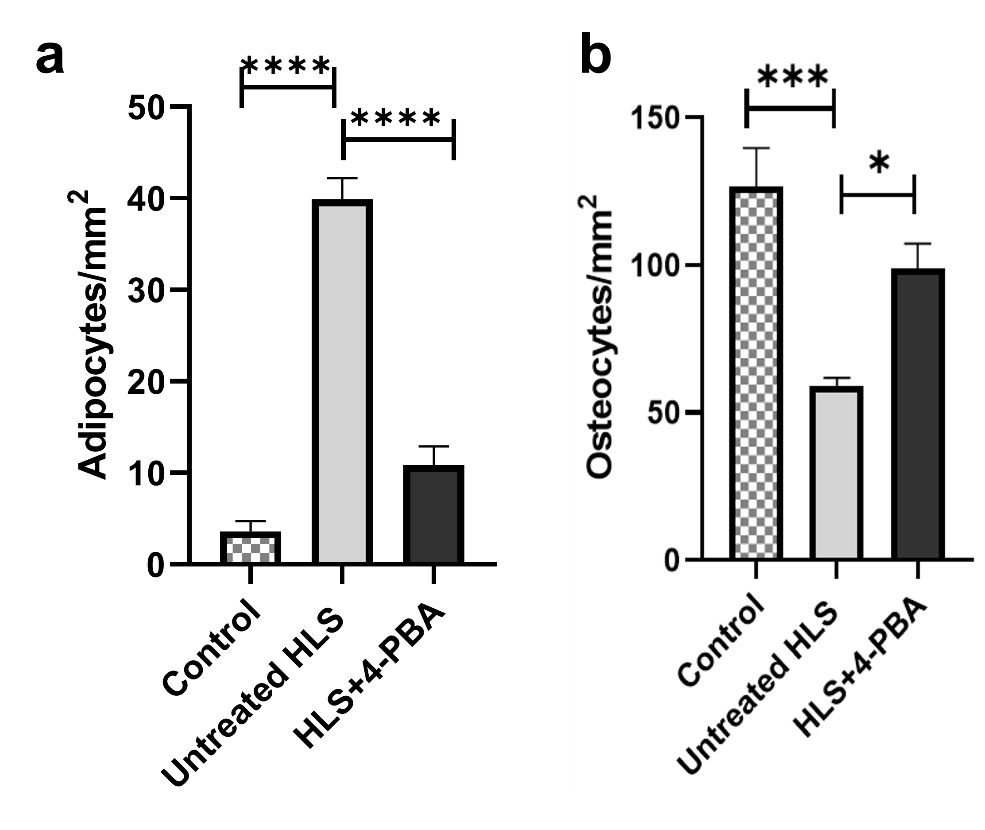
**

**Supplementary Fig 4. Quantification of number of adipocytes in bone marrow area and number of osteocytes in cortex of tibia among control, untreated HLS, and HLS+4-PBA treated mice.** (a) Quantification of the number of adipocytes per mm^2^ in the bone marrow area in tibia compared among the study groups and (b) Quantification of the number of osteocytes per mm^2^ in the diaphysis of tibial cortical bones among the among the study groups. Data are expressed as mean ± SEM. (n=3/group)(*p<0.05; ***p<0.001).

**
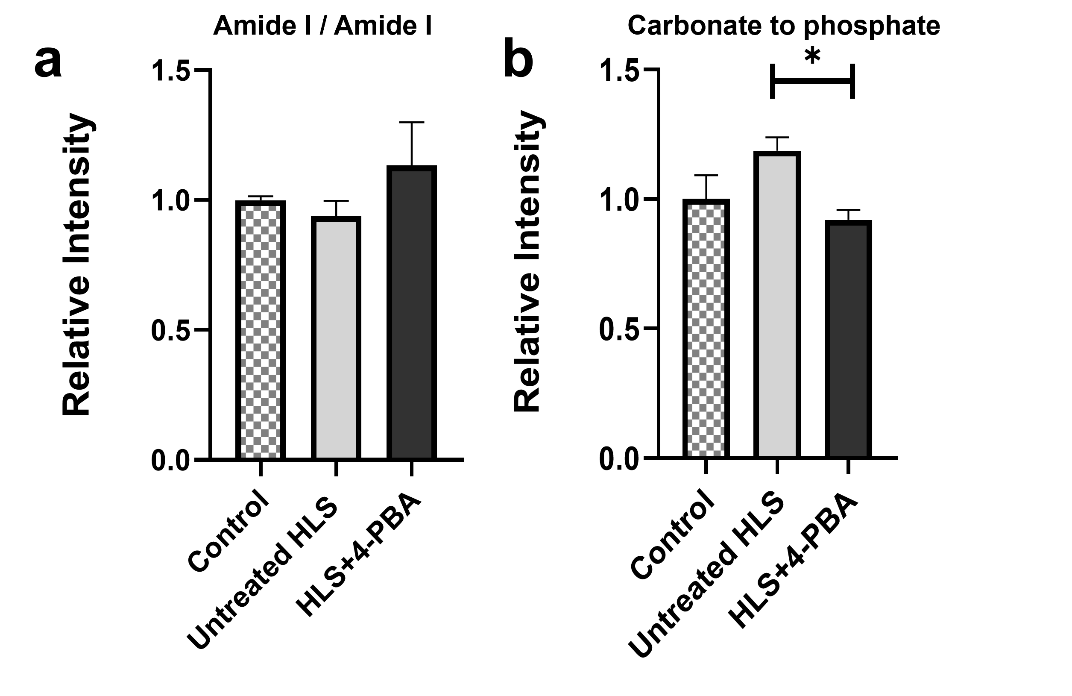
**

**Supplementary Fig 5. Raman spectra measurement of amide I/amide I crosslinking and carbonate to phosphate ratios among control, untreated HLS, and HLS+4-PBA treated mice**. (a) amide I/ amide I band ratio (1660 cm^-1^/1690 cm^-1^) and (b) carbonate to phosphate at (1070 cm^-1^/430 cm^-1^). Data are expressed as mean ± SEM. (n=5/group) (HLS; hindlimb suspension, 4-PBA; 4-phenyl butyrate). (*p<0.05)

**Supplementary Table 1.** Sequence of the primers used in the study.

| **Gene** | **Sequence** |
| --- | --- |
| Alkaline phosphatase | FP: TGTACCCGAAGAACAGAACCGAC  RP: CAGACCATCTAGCCTTGTACC |
| Osteocalcin | FP : TACACGTGCAGGTCAATCCC  RP : GGCGGTCTTCAAGCCATACT |
| TRAP | FP: GGCTACTTGCGGTTTCACTATG  RP: GGGAGGCTGGTCTTAAAGAGTG |
| Cathepsin K  Sclerostin  18S | FP: CAGCTTCCCCAAGATGTGAT  RP: AGCACCAACGAGAGGAGAAA  FP: AAGCCTTCAGGAATGATGCCA  RP: GAGGTCTGCCTCCATTCTCC  FP: GGAGAGGGAGCCTGAGAAAC  RP: CCTCCAATGGATCCTCGTTA |
